# Sex differences in interleukin‐6 stress responses in people with Type 2 diabetes

**DOI:** 10.1111/psyp.13334

**Published:** 2019-01-22

**Authors:** Laura Panagi, Lydia Poole, Ruth A. Hackett, Andrew Steptoe

**Affiliations:** ^1^ Research Department of Behavioural Science and Health University College London London United Kingdom

**Keywords:** interleukin‐6, laboratory stress, sex differences, stress responses, Type 2 diabetes

## Abstract

People with Type 2 diabetes (T2D) show dysregulated inflammatory responses to acute stress, but the effect of sex on inflammatory responses in T2D remains unclear. The purpose of this study was to investigate differences in interleukin (IL)‐6 stress responses between older men and women with T2D. One hundred and twenty‐one people (76 men; mean age = 64.09, *SD* = 7.35, 45 women; mean age = 63.20, *SD* = 6.70) with doctor‐verified T2D took part in this laboratory‐based stress testing study. Participants carried out acute mental stress tasks, and blood was sampled at baseline, immediately poststress, 45 min poststress, and 75 min poststress to detect plasma IL‐6 concentrations. IL‐6 change scores were computed as the difference between the baseline measurement and the three time points poststress. Main effects and interactions were tested using mixed model analysis of covariance. We found a significant main effect of time on IL‐6 levels, and a significant Sex × Time interaction. In adjusted analyses including the three change scores and all the covariates, the significant Sex × Time interaction was maintained; IL‐6 responses were greater in women at 45 and 75 min poststress compared with men, adjusting for age, body mass index, smoking, household income, glycated hemoglobin, oral antidiabetic medication, insulin/other injectable antidiabetic medication, depressive symptoms, and time of day of testing. Different inflammatory stress response pathways are present in men and women with T2D, with women producing larger IL‐6 increases. The long‐term implications of these differences need to be elucidated in future studies.

## Abstract 2

This laboratory study is the first to examine sex differences in inflammatory stress responsivity in people with Type 2 diabetes (T2D). Older women with T2D have higher rates of some inflammatory‐related conditions compared to their male counterparts, including coronary heart disease and stroke. Notably, these conditions are leading causes of premature mortality in this population. IL‐6 is adversely involved in diabetes progression, and we found evidence of sex differences in IL‐6 stress responses. This study contributes to the understanding of the mechanisms that may explain, to some extent, the different levels of disease vulnerability in this a priori high‐risk population.

## 1 INTRODUCTION

Psychosocial stress is a risk factor for major chronic conditions including obesity (Moore & Cunningham, 2012), Type 2 diabetes (T2D; Demakakos, Pierce, & Hardy, 2010; Nyberg et al., 2014), and coronary heart disease (CHD; Kivimaki & Steptoe, 2018). The stress‐T2D relationship is not fully explained by behavioral factors (Demakakos et al., 2010; Hackett & Steptoe, 2017; Nyberg et al., 2014), suggesting that stress‐related biological dysfunction may also be relevant.

Dysregulated biological processes can be manifested as dysregulated (re)activity of the stress biomarkers—either raised elevated resting/basal levels or maladaptive responses to acute stress (McEwen, 1998). Stress responsivity can be examined using a laboratory stress testing design (Gerin, 2010). More specifically, in the laboratory, stress biomarkers can be measured before, during, and after acute stressful tasks that are hypothesized to emulate real‐life stressors. This research strategy enables the control of environmental confounders, such as noise and room temperature, and is typically focused on dynamic changes in biomarkers from pre‐ to poststress that could not be detectable if single measures were taken (Gerin, 2010).

The immune system is typically involved with the acute stress response (Chrousos, 1998), and a considerable number of studies have examined inflammatory stress responses that are known to reflect the activation of the innate immune system (Marsland, Walsh, Lockwood, & John‐Henderson, 2017; Steptoe, Hamer, & Chida, 2007). The circulating proinflammatory cytokine interleukin (IL)‐6 is one of the most frequently measured markers in laboratory studies (Marsland et al., 2017). IL‐6 is produced from a number of cell types after stimulation by the proinflammatory cytokine IL‐1β, and in turn stimulates the synthesis of the acute phase C‐reactive protein (CRP) by hepatocytes (Steptoe et al., 2007). Results from two meta‐analyses showed that laboratory stress testing induces reliable increases in plasma IL‐6 (Marsland et al., 2017; Steptoe et al., 2007). Increases can be detectable in blood circulation as early as 10 min after stress and reach a peak around 90 min poststress (Marsland et al., 2017).

A very prominent feature of inflammatory stress responsivity is the large variability in the magnitude of stress responses between individuals. For example, increasing evidence from basic and clinical research shows that sex, as a fundamental biological factor and an intrinsic individual characteristic, is relevant to shaping the acute stress response. A 2017 review of animal studies demonstrated consistent differences in biological responses to acute stress between male and female subjects (Novais, Monteiro, Roque, Correia‐Neves, & Sousa, 2017). Human studies involving relatively healthy middle‐ or older‐aged participants also suggest marked sex differences in the magnitude of IL‐6 responses. In particular, three previous studies have provided evidence for women being more inflammatory responsive than men following laboratory stress. Specifically, women showed greater IL‐6 increases from baseline (prestress) to 30 min (Lockwood, Marsland, Cohen, & Gianaros, 2016), 45 min (Endrighi, Hamer, & Steptoe, 2016; Steptoe, Owen, Kunz‐Ebrecht, & Mohamed‐Ali, 2002), and 75 min (Endrighi et al., 2016) following stress (poststress).

Increased IL‐6 responsivity might be health damaging, conferring elevated risk for inflammatory‐related conditions. Albeit scarce, there is evidence that individuals who show increased IL‐6 responses in the laboratory are prone to developing cardiovascular disease (CVD) risk factors in the long term outside the laboratory environment. For example, in the study of Brydon and Steptoe (2005) of healthy middle‐aged participants, greater IL‐6 stress responses predicted larger increases in ambulatory systolic blood pressure 3 years later. Results were adjusted for baseline ambulatory blood pressure, acute blood pressure stress responses, age, sex, body mass index (BMI), and smoking. Moreover, hostility has been related to larger IL‐6 stress responses in people with T2D, indicating an association between unfavorable personality characteristics and more pronounced IL‐6 increases poststress (Hackett, Lazzarino, Carvalho, Hamer, & Steptoe, 2015). Finally, a recent study found that larger IL‐6 responses to acute stress in the laboratory are associated with higher levels of systemic inflammation in men, measured by resting CRP, possibly conferring higher risk for inflammatory‐related diseases (Lockwood et al., 2016).

T2D is a chronic metabolic disease prevalent in more than 3 million people in the UK (Holman, Young, & Gadsby, 2015). T2D has been characterized by physiological dysregulation across multiple biological systems, along with chronic life stress (Steptoe et al., 2014). For example, the IL‐6 profile of people with established T2D differs significantly from that of healthy individuals. In a 2013 meta‐analysis, a dose‐response relationship between IL‐6 and risk of new onset T2D was observed (Wang et al., 2013), suggesting that inflammatory dysregulation exists prior to diabetes diagnosis. Epidemiological evidence indicated that people with T2D have elevated basal IL‐6 concentrations compared to healthy controls (Pickup, 2004). In the laboratory environment, people with T2D have been found to have significantly higher plasma IL‐6 levels both pre‐ and poststress and showed smaller IL‐6 increases after stress compared to healthy controls. Therefore, despite diminished IL‐6 responses, absolute levels remained higher in the diabetes group (Steptoe et al., 2014). CVD is a major complication of T2D (Rao Kondapally Seshasai et al., 2011; Sarwar et al., 2010), and raised IL‐6 levels in established T2D have been associated with CVD risk and mortality, independently of other inflammatory markers such as fibrinogen or CRP (Lowe et al., 2014).

Given that IL‐6 is adversely involved in T2D and its related complications, sex differences in IL‐6 stress responsivity may reflect different degrees of susceptibility to T2D development and progression. Notably, before the age of the menopause, the incidence of T2D and atherosclerosis is higher in men compared with women, while after the menopause these conditions in women equal or exceed the rates observed in same‐aged men (Gubbels Bupp, 2015). Moreover, the impact of psychosocial stress on T2D risk is greater in women than in men (Kautzky‐Willer, Harreiter, & Pacini, 2016), and some inflammatory‐mediated conditions are more prevalent in women with T2D compared with their male counterparts, including CHD, stroke, kidney disease, fibromyalgia, depression, and vascular dementia (Anderson, Freedland, Clouse, & Lustman, 2001; Chatterjee et al., 2016; Kautzky‐Willer et al., 2016; Peters, Huxley, & Woodward, 2014a, 2014b; Yanmaz, Mert, & Korkmaz, 2012). The mechanisms that underpin these differences are likely, to some extent, the result of sex differences in stress‐related biology. Better understanding of the potentially protective effects of one sex could help to develop preventive and management strategies for both sexes.

To the best of our knowledge, no previous studies have examined sex differences in inflammatory stress responses in people with T2D. The aim of this study was to extend previous research in stress‐related biology between men and women with T2D. The objective of this study was to investigate potential differences in IL‐6 stress responses between older men and women with T2D. We hypothesized that women with T2D will show greater IL‐6 stress responses compared with men with T2D.

## 2 METHOD

### 2.1 Study sample

One hundred and forty people (88 men, 52 women) aged 50–75 years with doctor‐verified T2D diagnosis took part in a laboratory‐based stress testing study. This was part of a larger trial comparing biological responses to laboratory stress between healthy individuals and people with T2D, and full details of the sample can be found elsewhere (Steptoe et al., 2014). In order to detect small to moderate effect sizes (*δ* = 0.32, *p* < 0.05), we aimed to recruit at least 125 people. Participants were recruited from diabetes outpatient clinics and primary care practices in London between March 2011 and July 2012. Inclusion was limited to people without a history or previous diagnosis of CHD, inflammatory diseases, allergies, or mood disorders, and no evidence of autonomic neuropathy based on self‐report. Obesity is one of the main risk factors for diabetes (Guh et al., 2009); therefore, it was not possible to exclude obese individuals from this study. All participants gave fully informed written consent to take part in the study, and ethical approval was obtained by the UK National Research Ethics Service.

Out of 140 participants, 34 had missing data for at least one IL‐6 measurement owing to issues in blood sampling and analysis. More precisely, as the majority of participants were obese, maintaining a functioning cannula for the duration of the laboratory session presented technical challenges; the cannula failed partway through the laboratory procedure for some participants. We had a protocol to not reattempt blood draw in this case so as to minimize distress for participants. A sensitivity analysis was also carried out to check for significant differences in participants’ characteristics between those with full data on IL‐6 and those with missing data on at least one IL‐6 measurement using *t* tests for continuous and chi‐square for categorical variables. This analysis revealed that participants of higher BMI were more likely to have missing data on at least one IL‐6 measurement (*p* = 0.017), but there were no other differences between those with and without missing data on IL‐6.

### 2.2 Procedure

Participants were invited for individual stress testing in our light‐ and temperature‐controlled laboratory at University College London (UCL). Testing was performed in either the morning or afternoon and was based on a standard protocol previously used in the same laboratory (Hamer, O’Donnell, Lahiri, & Steptoe, 2010; Steptoe, Owen et al., 2002). Pretesting instructions included to avoid taking any anti‐inflammatory or antihistamine medication up to 7 days before testing session, to refrain from performing vigorous exercise and consuming alcohol from the evening prior to testing, and to avoid caffeinated beverages or smoking for at least 2 hr before testing. Participants who reported colds or other infections on the day of testing were rescheduled.

On the testing day, participants’ anthropometric characteristics (height, weight, waist, hip, percentage of body fat) were first assessed using standardized techniques. Following this, a venous cannula was inserted into participants’ forearm for blood sample collection. Within the last 5 min of a 30‐min resting phase, a blood sample was drawn to detect baseline IL‐6 levels. We then administered two 5‐min mental stress tasks, and blood was sampled again immediately after the tasks (immediately post‐task measurement), at 45 min (45 min post‐task measurement), and at 75 min after the completion of the tasks (75 min post‐task measurements). Blood samples were collected using ethylenediaminetetraacetic acid (EDTA) tubes (four tubes for each participant), which were centrifuged immediately after collection at 2,500 rpm for 10 min at room temperature. Ten minutes later, plasma was removed from the tubes, aliquoted into 0.5‐ml portions, and stored at −80°C until batch analysis at a later date. For batch analysis, we used Quantikine high sensitivity two‐site enzyme‐linked immunosorbent assay (ELISA) from R&D Systems (Oxford, UK). IL‐6 sensitivity ranged between 0.016 and 0.110 pg/ml, and the mean intra‐assay and interassay coefficients of variations were 7.3% and 7.7%, respectively.

### 2.3 Mental stress tasks

Two 5‐min mental stress tasks were administered in random order: a computerized version of the Stroop color‐word interference task and the mirror tracing task. The Stroop task requires successive reporting of target color words (e.g., blue, red) presented (on a screen) in an incongruous color. The mirror tracing task requires participants to move a metal stylus to trace a star while looking at the mirror image. When the stylus comes off the star’s outer line, a loud noise is emitted by the device and a mistake is counted (Lafayette Instruments Company, Lafayette, IN). We told participants that the average person achieves five tracings with a minimum of mistakes in the time given. These tasks are used widely in experimental studies as they induce robust biological stress responses, and they have been used in previous studies by our group (Hamer et al., 2010; Steptoe, Feldman et al., 2002).

### 2.4 Study measures

#### 2.4.1 Predictor variable

##### Sex

Self‐reported information on sex was obtained, categorized into man/woman.

#### 2.4.2 Dependent variable

##### IL‐6 stress responses

Plasma IL‐6 concentrations were measured at four time points: baseline, immediately post‐task, 45 min post‐task, and 75 min post‐task. Three IL‐6 mean change/delta (Δ) scores were used as dependent variables, reflecting the mean difference/change in IL‐6 from baseline to post‐task measurement: immediately post‐task minus baseline (ΔIL‐6 immediately post‐task), 45 min post‐task minus baseline (ΔIL‐6 45 min), and 75 min post‐task minus baseline (ΔIL‐6 75 min). Higher positive delta scores indicated greater IL‐6 increases from baseline to post‐task measurements.

#### 2.4.3 Covariates

We selected a number of covariates based on previous research that has indicated their influence on inflammatory (re)activity: age (Steptoe, Owen et al., 2002), BMI (kg/m^2^; McInnis et al., 2014), smoking status (smoker/nonsmoker; Marsland et al., 2017), household income (<£20,000/£20,000 – £40,000/£40,000 – £60,000/> £60,000) as an indicator of socioeconomic status (Steptoe, Owen et al., 2002; Stringhini et al., 2013), and depressive symptoms (Howren, Lamkin, & Suls, 2009). Presence of a health condition (healthy vs. having a clinical condition) is known to influence inflammatory stress responses (Steptoe et al., 2007); thus, glycated hemoglobin (HbA1c) and antidiabetic medication (oral medication/insulin or other injectable medication) at the time of testing were also selected as these could reflect different levels of disease severity. IL‐6 shows some diurnal variation with increasing levels over the course of the day (Vgontzas et al., 2005); therefore, time of testing (am/pm) was also included in the models.

Age, smoking status, and household income were recorded by self‐report, and BMI was calculated from height and weight measurements on the day of testing. Antidiabetic medication was also recorded by self‐report and confirmed by inspection of medication packaging on the day of testing. HbA1c was assessed from the baseline blood draw using standardized techniques. For depressive symptoms, participants completed the Centre of Epidemiological Studies–Depression (CES‐D) scale (Radloff, 1977). This is a standardized questionnaire consisting of 20 items relating to depressive symptoms over the previous week. Responses can range from 0 (*rarely or none of the time/less than 1 day*) to 3 (*most or all the time/5–7 days*). Responses were summed with higher scores indicating greater depressive symptoms over the previous week. The Cronbach’s alpha was satisfactory in this sample (0.86).

#### 2.4.4 Other measures

Other variables considered in secondary analyses were ethnicity (white/Asian/Afro‐Caribbean/other), educational level (no qualifications or elementary school diploma/up to O levels or middle or junior high school diploma/A levels–ONC or high school or senior high school diploma**/**degree, or university undergraduate certificate or above**)**, marital status (married/single/separated, or divorced or widowed), and hours of moderate or vigorous physical activity per week were recorded based on self‐report. Further adiposity measures were also recorded: percentage of body fat and waist‐to‐hip ratio. Adiposity measures were calculated from measurements taken on the testing day. Cardiovascular medication at the time of testing was also recorded by self‐report and confirmed by inspection of medication packaging on the day of testing. Categories of cardiovascular medication included antihypertensive medication (yes/no), beta‐blockers (yes/no), cholesterol‐lowering drugs (yes/no), and aspirin (yes/no).

Baseline subjective stress and task perception variables were also measured in this study. Baseline subjective stress was measured before the tasks by asking participants, “How stressed do you feel at the moment?” Participants answered on a 7‐point scale, with higher scores indicating greater baseline subjective stress. After each of the stress tasks, participants were asked to rate how stressful the task was using a 7‐point scale (1 = *not at all stressful*, 7 = *very stressful*). Moreover, ratings of task involvement, task control, task performance, and task difficulty were also taken after each of the tasks, with responses on a 7‐point scale (1 = *not at all involved, in control, well, difficult*; 7 = *very involved, in control, well, difficult*). Responses for the two tasks were averaged to create overall scores for task perceptions. Higher scores indicated higher stress, higher involvement, higher control, better performance, and greater difficulty, respectively.

### 2.5 Statistical analysis

IL‐6 values were skewed, thus log‐*n* transformation was used in all analyses except values presented in Table 1 for ease of interpretation. Characteristics of men and women were compared with chi‐square for categorical and *t* tests for continuous variables.

**Table 1:** Sample characteristics by sex

Characteristic	Men (*n* = 76)	Women (*n* = 45)	*p* a
Age, *M* (*SD*) years	64.09 (7.35)	63.20 (6.70)	0.506
Marital status, *n* (%)			0.070
Married	45 (59.2)	17 (37.8)	
Single	13 (17.1)	13 (28.9)	
Divorced, separated, or widowed	18 (23.7)	15 (33.3)	
Household income, *n* (%)			0.060
<£20,000	31 (40.8)	21 (46.7)	
£20,000–£40,000	17 (22.4)	17 (37.8)	
£40,000–£60,000	8 (10.5)	3 (6.7)	
> £60,000	20 (26.3)	4 (8.9)	
Ethnicity, *n* (%)			0.021
White	61 (80.3)	35 (77.8)	
Asian	10 (13.2)	1 (2.2)	
Afro‐Caribbean	4 (5.3)	4 (8.9)	
Other	1 (1.3)	5 (11.1)	
Educational level, *n* (%)^b^			0.726
No qualifications (elementary school diploma)	5 (6.8)	3 (6.7)	
Up to O levels (middle or junior high school diploma)	16 (21.6)	6 (13.3)	
A levels/ONC (high school or senior high school diploma)	7 (9.5)	5 (11.1)	
Degree (university undergraduate certificate) or above	46 (62.2)	31 (68.9)	
Smoking, *n* (%) smoker	11 (14.5)	5 (11.1)	0.803
Moderate/vigorous physical activity, *M* (*SD*) hours/week^c^	4.08 (8.19)	3.89 (4.18)	0.889
BMI, *M* (*SD*) kg/m^2^	30.11 (5.13)	32.14 (6.33)	0.057
Body fat, *M* (*SD*) %	30.96 (4.58)	44.32 (6.94)	<0.001
Waist‐to‐hip ratio, *M* (*SD*) cm^d^	1.01 (0.07)	0.97 (0.11)	0.050
HbA1c, *M* (*SD*) %	7.28 (1.36)	7.38 (1.64)	0.700
Oral antidiabetic medication, *n* (%) yes	62 (81.6)	38 (84.4)	0.878
Insulin/other injectable diabetic medication, *n* (%) yes	9 (11.8)	6 (13.3)	1.000
Antihypertensive medication, *n* (%) yes	55 (72.4)	30 (66.7)	0.647
Beta‐blockers, *n* (%) yes	7 (9.2)	4 (8.9)	1.000
Cholesterol‐lowering drugs, *n* (%) yes	57 (75.0%)	37 (82.2)	0.486
Aspirin, *n* (%) yes	33 (43.4)	9 (20.0)	0.016
CES‐D scale, *M* (*SD*)	11.60 (8.19)	11.84 (8.28)	0.875
Baseline subjective stress, *M* (*SD*)	1.45 (0.82)	1.58 (1.01)	0.441
Task stress, *M* (*SD*)	4.25 (1.50)	5.00 (1.33)	0.006
Task involvement, *M* (*SD*)	5.24 (1.52)	5.66 (1.59)	0.153
Task control, *M* (*SD*)	2.50 (1.21)	2.43 (1.47)	0.798
Task performance, *M* (*SD*)	2.23 (1.17)	2.13 (1.31)	0.674
Task difficulty, *M* (*SD*)	5.63 (1.07)	5.99 (1.00)	0.074
Baseline IL‐6 (unlogged values; pg/mL)^e^	2.19 (1.29)	2.01 (1.31)	0.459
IL‐6 immediately post‐task (unlogged values; pg/mL)^f^	2.21 (1.22)	2.14 (1.38)	0.785
IL‐6 45 min post‐task (unlogged values; pg/mL)^g^	2.32 (1.41)	2.33 (1.59)	0.972
IL‐6 75 min post‐task (unlogged values; pg/mL)^h^	2.30 (1.18)	2.26 (1.38)	0.873
AM (morning) testing, *n* (%) yes	32 (42.1)	21 (46.7)	0.765

Note

*N* = 121. AM = after midnight; BMI = body mass index; CES‐D = Centre for Epidemiological Studies–Depression; HbA1c = glycated hemoglobin; kg/m^2^ = kilogram/square meter; *M* = mean; *N* = number; *n* = number; ONC = Ordinary National Certificate; pg/mL = pictogram/millilitre; *SD* = standard deviation.

a Differences by sex were checked using *t *tests for continuous variables and chi‐square tests for categorical variables.

b *n* = 119.

c *n* = 111.

d *n* = 120.

e *n* = 116.

f *n* = 112.

g *n* = 101.

h *n* = 93.

Significant main effects and interactions were first tested using mixed model analysis of variance (ANOVA). IL‐6 values were analyzed across four time points: baseline, immediately post‐task, 45 min post‐task, and 75 min post‐task, with sex being the between‐subjects factor and the four time points the within‐subject factor. Significant interactions were further explored using mixed model analysis of covariance (ANCOVA). In this model, three IL‐6 mean change scores were used as dependent variables (values were first logged and then calculated to change scores), reflecting the mean difference in IL‐6 from baseline to immediately post‐task (ΔIL‐6 immediately post‐task), 45 min post‐task (ΔIL‐6 45 min), and 75 min post‐task (ΔIL‐6 75 min). Higher positive delta scores indicate greater IL‐6 increases from baseline to post‐task measurements. To avoid overadjustment, only nine covariates were included: age, BMI, smoking status, household income, HbA1c, oral antidiabetic medication, insulin/other injectable antidiabetic medication, depressive symptoms, and time of testing. Change scores account for baseline levels; therefore, the three change scores rather than the four time points were used as dependent variables in the main analysis. Secondary analyses were carried out to control for potential confounders using mixed model ANCOVA. Moreover, secondary analyses were conducted to test for the effects of age on IL‐6 responsivity in women using mixed model and repeated measures ANOVA.

We analyzed data from 121 participants (76 men, 45 women) who provided data on sex and all covariates. Results in Table 1 are presented as means and standard deviations (*M* ± *SD*) for continuous variables or *n* and per cent for categorical variables. The level of significance was set at *p < *0.05, though exact *p* values are reported throughout. Statistical analyses were conducted using SPSS version 24 (SPSS, Chicago, IL).

## 3 RESULTS

### 3.1 Sample characteristics by sex

Table 1 presents sample characteristics by sex. There were no significant sex differences in age, marital status, income, or education. There was a sex difference in ethnicity, with more men than women being of South Asian origin, *χ* ^2^(3) = 9.77, *p* = 0.021, *V* = 0.28. Participants were obese on average. Women had significantly higher body fat percentage, *t*(66.977) = −11.51, *p < *0.001, *d* = 2.27, 95% CI [−15.67, −11.04], while men had marginally higher waist‐to‐hip ratios, *t*(118) = 1.98, *p* = 0.050, *d* = 0.36, 95% CI [−0.00, 0.06]. Men and women did not differ in diabetes characteristics or lifestyle variables including HbA1c, antidiabetic medication, BMI, smoking, or physical activity, though men were more likely to take aspirin at the time of testing; *χ* ^2^(1) = 5.85, *p* = 0.016, *φ *= −0.24. No other sex differences were found (Table 1).

### 3.2 Sex differences in perceived tasks appraisals

Task ratings showed that women perceived the tasks as more stressful than men, *t*(188) = −2.78, *p* = 0.006, *d* = 0.53, 95% CI [−1.29, −0.22]. There were no significant sex differences in task difficulty, task involvement, task control, or task performance (Table 1).

### 3.3 Sex differences in IL‐6 stress responses

**Figure 1:**
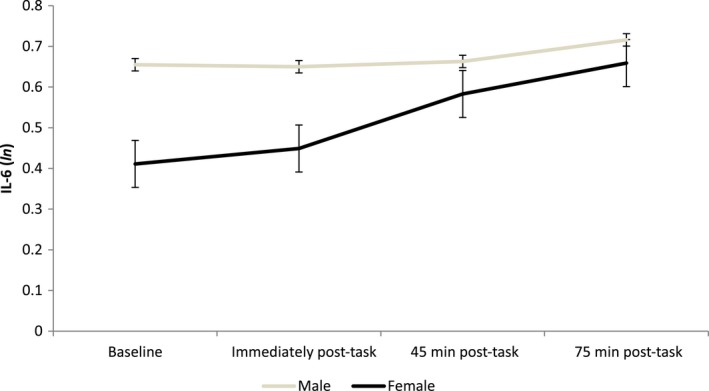
Mean plasma IL‐6 values at four time points in men and women with T2D (*n* = 92). Values are unadjusted mean plasma IL‐6 (logged *n*) at baseline, immediately post‐task, 45 min, and 75 min post‐task. Pairwise comparisons showed significant differences within women category at 45 and 75 min compared to baseline (45 min compared to baseline: *p < *0.001, 95% CI [0.094, 0.249], 75 min compared to baseline: *p* < 0.001, 95% CI [0.140, 0.355]) and immediately post‐task values (45 min compared to immediately post‐task: *p* = 0.002, 95% CI [0.052, 0.216], 75 min compared to immediately post‐task: *p* < 0.001, 95% CI [0.108, 0.311]). Marginally significant increases were observed within men category at 75 min compared to immediately post‐task (*p* = 0.087, 95% CI [−0.010, 0.142]) and 45 min values (*p* = 0.088, 95% CI [−0.008, 0.113]). There were no significant sex differences in baseline or post‐task IL‐6 values between women and men (checked with independent samples *t* tests). Error bars are standard errors of the mean. IL‐6 = interleukin 6; *ln* = log *n*

Plasma IL‐6 levels increased significantly over time (time effect, *F*(2.436, 219.279) = 13.18, *p < *0.001, $\eta_{p}^{2}$ = 0.128), from baseline ($\overset{¯}{x}$ = 0.53 ± 0.06) to 75 min ($\overset{¯}{x}$ = 0.69 ± 0.06) poststress. There was also a significant Sex × Time interaction, *F*(2.436, 219.279) = 5.39, *p* = 0.003, $\eta_{p}^{2}$ = 0.056 (Figure 1), providing evidence of sex differences in IL‐6 stress responses.

**Figure 2:**
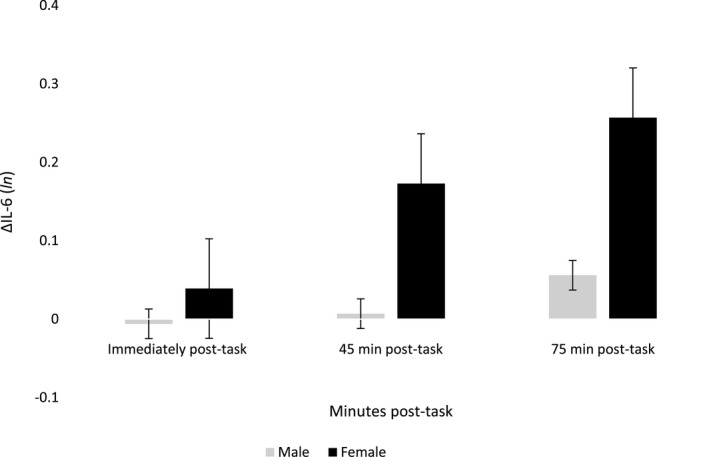
Mean changes in plasma IL‐6 from baseline to three time points post‐task in men and women with T2D (*n* = 92). Results are adjusted for age, BMI, smoking status, household income, HbA1c, oral antidiabetic medication, insulin/other injectable drugs use, depression symptoms, and time of testing. Error bars are standard errors of the mean. ΔIL‐6 = delta interleukin‐6 (change score); *ln* = log *n*

The ANCOVA model including the three change scores as the within‐subject factor and sex as the between‐subjects factor revealed a significant Sex × Time interaction indicating that IL‐6 responses differed significantly between men and women after controlling for age, BMI, smoking, income, HbA1c, oral antidiabetic medication, insulin/other injectable antidiabetic medication, depressive symptoms, and time of testing, *F*(1.881, 152.384) = 4.19, *p* = 0.019, $\eta_{p}^{2}$ = 0.049. Significant differences between women and men occurred at 45 min when women exhibited greater IL‐6 responses compared with men (ΔIL6 45 min for women: $\overset{¯}{x}$ = 0.17 ± 0.04 vs. men: $\overset{¯}{x}$ = 0.007 ± 0.03, *p* = 0.002, 95% CI [0.65, 0.27]), and this difference was sustained at 75 min responses (ΔIL6 75 min for women: $\overset{¯}{x}$ = 0.26 ± 0.06 vs. men: $\overset{¯}{x}$ = 0.06 ± 0.04, *p* = 0.004, 95% CI [0.06, 0.34]). The adjusted IL‐6 stress responses are depicted in Figure 2.

Using a secondary model, analyses were carried out to control for the effects of body fat percentage, waist‐to‐hip ratio, aspirin intake, ethnicity, and stress perception, and the significant Sex × Time interaction was maintained, *F*(1.776, 147.386) = 4.05, *p* = 0.023, $\eta_{p}^{2}$ = 0.047. Despite being not statistically significant, baseline IL‐6 levels were slightly higher for men than women (as reflected in Figure 1). Adjusting for baseline IL‐6 levels attenuated the Sex × Time interaction (*p* = 0.075).

We carried out additional analysis to test for the effects of age in the magnitude of IL‐6 stress responses in women. Within women, IL‐6 levels over time did not interact with age (*p* = 0.955). Examining IL‐6 levels over time separately for younger and older women (≤ 55/> 55) revealed a greater main effect of time for older women, *F*(3, 21) = 4.74, *p* = 0.011, versus younger women, *F*(2.083, 60.412) = 8.88, *p* < 0001.

## 4 DISCUSSION

We investigated sex differences in IL‐6 responses to laboratory stress in a sample of middle‐and older‐aged men and women with T2D. Although the mean IL‐6 values were higher for men at baseline, this difference was not significant. Female participants exhibited significantly larger IL‐6 stress responses at 45 and 75 min post‐task compared to male participants. IL‐6 increases in women were detectable at 45 min post‐task, and continued to increase reaching the highest levels at 75 min post‐task. IL‐6 changes in men were more delayed, showing observable, albeit marginally significant increases at 75 min post‐task. Significant sex differences in IL‐6 responses were independent of age, BMI, smoking status, household income, HbA1c, oral antidiabetic medication, insulin/other injectable antidiabetic medication, depressive symptoms, and time of testing. Secondary analysis confirmed the Sex × Time interaction after controlling for body fat percentage, waist‐to‐hip ratio, aspirin intake, ethnicity, and stress perception. These findings highlight different inflammatory response pathways to acute stress between men and women with T2D.

The lack of previous studies in people with T2D or prediabetes does not permit direct comparisons with similar samples. Nevertheless, our findings are consistent with three previous studies on healthy middle‐ or older‐aged participants, which demonstrated greater IL‐6 stress responses in women (Endrighi et al., 2016; Lockwood et al., 2016; Steptoe, Owen et al., 2002). On the contrary, null and conflicting findings have been reported in studies of younger participants (Edwards, Burns, Ring, & Carroll, 2006; Rohleder, Schommer, Hellhammer, Engel, & Kirschbaum, 2001), although the smaller sample sizes in these studies (40 and 45 participants, respectively) may have contributed to the inconsistent findings.

It is also plausible that lower levels of reproductive hormones associated with ageing in women or menopausal status may explain the sex differences that are more consistently found in older participants. In the study of Endrighi and colleagues (2016), circulating IL‐6 stress responses were greater in women compared to men, with all women being postmenopausal. Additionally, in the study of Prather and colleagues (2009), lipopolysaccharide‐stimulated IL‐6 production was significantly greater in postmenopausal women at 30 min poststress compared with men and premenopausal women. Reproductive hormones may have contributed to sex differences found in our study. Estrogens, which are thought to have anti‐inflammatory effects, are reduced in ageing women (Winters, 2001), potentially having a permissive effect on IL‐6 production and gene expression. We did not collect information on menopausal status in our study; therefore, it was not possible to directly test this hypothesis, though menopause usually occurs between 45 and 55 years of age and the mean age for a woman to reach the menopause in the UK is 51 (NHS, 2018). Mean age of the women in our sample was 63 years old (*SD *= 6.70). Only three out of the 45 women in this study were ≤51 years old and eight of them were ≤55 years old. Thus, it is conceivable that the great majority of women in our sample were postmenopause. In our secondary analyses, we found a greater main effect of time on IL‐6 levels for older versus younger women. These findings lend support for the hypothesis that postmenopausal women may exhibit greater responses, but this needs testing directly in future studies.

Men and women in our study did not differ in key behavioral or clinical measures including smoking, physical activity, BMI, HbA1c, antidiabetic or cardiovascular medication. We found significant differences in aspirin use and body fat percentage, but including these factors in our secondary model did not attenuate the significant Sex × Time interaction effect. Women rated the tasks as more stressful than men, and it is possible that the effect of the stress tasks on participants’ mood was reflected in physiological activation. A laboratory‐based study with middle‐aged, healthy participants showed that task‐related increases in anxiety predicted increases in IL‐6 concentrations (Carroll et al., 2011). Nevertheless, including stress perception in our secondary model did not alter the significant Sex × Time interaction, and the Stress Perception × Time interaction was not significant. In the recent meta‐analysis of Marsland et al. (2017), the magnitude of IL‐6 responsivity did not vary as a function of task type (social threat vs. other stressor), but analyses were not conducted separately for women and men. Therefore, it is possible that tasks different than those used in the current study could lead to different stress appraisals and/or different inflammatory activation patterns between men and women. Another possible explanation is that sex differences in physiological reactivity reflect different levels of chronic life stress exposure between men and women with T2D. Depressive symptoms did not differ between men and women, though, and IL‐6 response differences were sustained despite inclusion of depressive symptoms in the analyses. Furthermore, human studies on sex differences in cortisol responsivity (cortisol is a glucocorticoid [GC] and a known anti‐inflammatory factor primarily involved in the acute stress response [Nicolaides, Kyratzi, Lamprokostopoulou, Chrousos, & Charmandari, 2015]) would be informative, but results on sex differences in cortisol responsivity appear inconsistent (Kudielka & Kirschbaum, 2005; Liu et al., 2017; Paris et al., 2010). Interestingly, there is evidence that the sensitivity of immune cells to the anti‐inflammatory effects of GCs increases 1 hr after acute stress only in men and not women (Rohleder et al., 2001). Nevertheless, it is not known how far these neuroendocrine and cellular sensitivity differences are generalized to people with diabetes. Stress‐related mediators, including IL‐6, cortisol, and catecholamines, act synergistically to maintain homeostasis (Karatsoreos & McEwen, 2010), and future studies need to examine how the interaction of these factors may influence sex differences in stress responsivity in people with T2D.

The clinical significance of heightened IL‐6 responses to stress remains unclear. In this study, although baseline sex differences in IL‐6 were not significant, IL‐6 was somewhat higher in men both at baseline and following stress. So, despite greater IL‐6 responses in women, female participants did not have greater IL‐6 concentrations than male participants overall. It is not certain whether the heightened stress responsivity of women or the higher absolute values among men is more hazardous to health. Interestingly, ageing women with T2D seem to be more vulnerable to psychosocial stress‐related factors and to inflammatory‐related clinical outcomes compared with similarly aged men (Kautzky‐Willer et al., 2016). It is plausible that sex differences in acute stress responsivity account for these sex differences. In previous laboratory studies, larger inflammatory responses were prospectively associated with CVD risk factors (Brydon & Steptoe, 2005; Ellins et al., 2008; Steptoe, Kivimaki, Lowe, Rumley, & Hamer, 2016). Nevertheless, due to the lack of prospective studies linking stress responses with physical health outcomes in people with T2D, it is not yet known whether heightened IL‐6 responsivity to stress is health damaging in T2D, as observed in healthy samples, since endothelial dysfunction and biological dysregulation are already established in this population (Ghiadoni et al., 2000; Steptoe et al., 2014). Studies investigating the longitudinal association between inflammatory stress responsivity and health outcomes in people with T2D are warranted. Finally, although the inflammatory response has been recognized as an integral part of the stress response (Chrousos, 1998; Marsland et al., 2017; Steptoe et al., 2007), IL‐6 is secreted from multiple sources, such as the liver (Heinrich, Castell, & Andus, 1990) or from muscle tissue during exercise (Petersen, 2012; Shephard, 2002), and these responses are not part of the inflammatory response. However, increases in IL‐6 and other pro‐ or anti‐inflammatory factors, such as monocyte chemoattractant protein (MCP)‐1 or IL‐1 receptor antagonist (IL‐1ra), have been reported in individual laboratory studies (Hackett, Hamer, Endrighi, Brydon, & Steptoe, 2012; Steptoe, Willemsen, Owen, Flower, & Mohamed‐Ali, 2001), suggesting a simultaneous upregulation of inflammatory factors, thus supporting the notion that IL‐6 activation represents changes within the inflammatory signaling cascade.

### 4.1 Study strengths and limitations

To the best of our knowledge, this is the first study to examine sex differences in IL‐6 stress responses in people with T2D. This is a relatively large laboratory study. However, full biological data were collected for only about three quarters of participants due to difficulties in blood sampling. We encountered practical challenges related to obesity during the insertion of the cannula in some participants, and therefore we controlled for BMI in all our analyses. Despite having a relatively long post‐task blood sampling period, a more extended sampling would have provided additional detail, particularly in view of evidence that IL‐6 responses to stress may continue to increase beyond 75 min after the tasks (Marsland et al., 2017). We took into account a wide range of potential confounders, including sociodemographic, behavioral, clinical, and psychological factors. The sole focus on IL‐6 provides a limited perspective on sex differences in stress‐related changes in inflammatory functioning. Nevertheless, we recently showed that other inflammatory factors, including IL‐1ra and MCP‐1, do not increase in response to acute stress in people with T2D, in contrast to IL‐6 (Panagi, Poole, Hackett, & Steptoe, 2018), adding value to testing IL‐6 responsivity in people with T2D. Despite not being statistically significant, baseline IL‐6 levels were slightly higher for men than women. It is possible that ceiling effects were involved in the change in IL‐6 over time for men. Indeed, adjustment for baseline IL‐6 in models attenuated the Sex × Time interaction. This possibility needs to be investigated in future studies with a larger sample size. Given the differences between people with T2D and healthy individuals in IL‐6 (re)activity, a direct comparison between people with T2D and healthy people would be of interest in future work. The participants of this study were middle‐aged and older men and women with T2D and without a history of CHD. They were recruited from the London area, and most of them were of white European ethnicity, thus we do not know whether similar patterns would emerge among other cohorts.

In conclusion, women with T2D exhibit greater stress‐induced increases in plasma IL‐6 than men, adjusting for covariates. The long‐term effects of these response patterns upon health need to be determined in future studies.
